# Molecular variants of the ATM gene in Hodgkin's disease in children

**DOI:** 10.1038/sj.bjc.6601522

**Published:** 2004-01-20

**Authors:** E Liberzon, S Avigad, I Yaniv, B Stark, G Avrahami, Y Goshen, R Zaizov

**Affiliations:** 1 Molecular Oncology, Felsenstein Medical Research Center, Petah Tikva, Sackler Faculty of Medicine, Tel Aviv University, Tel Aviv, Israel; 2 Pediatric Hematology Oncology, Schneider Children's Medical Center of Israel, Petah Tikva, Sackler Faculty of Medicine, Tel Aviv University, Tel Aviv, Israel

**Keywords:** ATM, Hodgkin lymphoma, mutations

## Abstract

Ataxia telangiectasia is an autosomal recessive disease with a striking predisposition of lymphoid malignancies. ATM mutations have been reported in adult sporadic lymphoma and leukaemia. The aim of this study was to investigate the possible involvement of the ATM gene in the carcinogenesis of Hodgkin disease in children. Tumours were obtained from 23 patients and were subjected to mutation screening and loss of heterozygosity analysis. Eight base substitutions were identified in seven patients. Of them, Y54Y, a silent change, was observed in two patients and a known polymorphism, D1853N, in three patients. Of the other two patients, one harboured a combined genotype P604S/F1463C, identified previously in two patients with Hodgkin lymphoma, and the other a novel missense mutation, V595A. The alterations were present in the germ line, and both had a more aggressive disease. In all, 100 matched normal ethnic controls were screened for these mutations and P604S/F1463C was identified in one healthy control. Loss of heterozygosity was identified in four patients and in three of them it was located centromeric to the ATM gene, and, in one, it spanned a large region, indicating the involvement of other tumour-suppressor genes in this disease. Missense variants of the ATM gene are a rare event in childhood Hodgkin disease.

Ataxia telangiectasia (A-T) is an autosomal recessive disorder characterised by telangiectasia, progressive ataxia, pulmonary infections, radiosensitivity and a combined immunodeficiency (Shiloh, 1995). Ataxia telangiectasia patients show an increased genomic instability, which results in translocations and inversions, leading to 100-fold cancer excess, particularly lymphoid malignancies (Morrell *et al*, 1986; Taylor *et al*, 1996). The most common lymphoid neoplasms are non-Hodgkin lymphomas, followed by acute lymphoblastic leukaemia and Hodgkin disease (5–10%) (Hecht and Hecht, 1990).

The ATM gene is responsible for A-T, located on chromosome 11q22–23, and encodes for serine–threonine kinase belonging to the phosphatidylinositol 3-kinase (PI3-K) family, and plays a central role in signalling pathways activated by DNA damage (Savitsky *et al*, 1995a, 1995b). Different studies identified a number of ATM targets including c-abl, TP53, CHK-2, NBS1 and BRCA1 (Shiloh, 2003).

Ataxia telangiectasia heterozygotes have been suggested to have a reduced life expectancy owing to a higher frequency of ischaemic heart disease (Su and Swift, 2000) and an increased risk for cancer, particularly for carcinoma of the breast (Swift *et al*, 1991; Broeks *et al*, 2000; Olsen *et al*, 2001). Several studies have indicated that ATM may also be involved in the development of some subtypes of sporadic lymphoma and leukaemia. Missense and truncation mutations in the ATM gene have been demonstrated in adult leukaemias: T-cell prolymphocytic leukaemia (T-PLL) (Stilgenbauer *et al*, 1997; Vorechovsky *et al*, 1997; Stoppa-Lyonnet *et al*, 1998; Yuille *et al*, 1998), and B-cell chronic lymphocytic leukaemia (B-CLL) (Bullrich *et al*, 1999; Schaffner *et al*, 1999; Stankovic *et al*, 1999; 2002). In adult lymphomas, Vorechovsky *et al* (1997) first demonstrated two missense variants in 32 non-Hodgkin lymphomas. In addition, missense and loss-of-function mutations were identified in mantle cell lymphoma (MCL), mainly associated with 11q22–23 loss of heterozygosity (LOH) (Stilgenbauer *et al*, 1999; Schaffner *et al*, 2000; Camacho *et al*, 2002). ATM mutations have been reported in diffuse large B-cell lymphoma (DLBCL) and follicular lymphoma (FL) (Gronbaek *et al*, 2002). Recently, Offit *et al* (2002) reported that ATM variants are rare in Hodgkin lymphoma and in radiation-induced breast cancer after treatment for Hodgkin disease.

The aim of the study was to investigate the possible involvement of ATM gene in the pathogenesis of Hodgkin lymphoma in children.

## MATERIALS AND METHODS

A total of 23 children with Hodgkin's disease participated in the study that was approved by the ethics committee of the hospital. Tumours were obtained from patients at diagnosis treated at the Center for Pediatric Hematology Oncology, Schneider Children’s Medical Center of Israel, between 1988 and 2001, and were snap-frozen in liquid nitrogen. The cohort consisted of 13 male and 10 female patients, ranging in age from 4.5 to 17.5 years (median 13 years). The tumours were classified according to the criteria established by Lukes and Butler (1966). The tumours belonged to the histological subtypes as follows: 19 nodullar sclerosis (NS) and four mixed cellularity (MC).

Total RNA was extracted from the tumours using the TRI-Reagent (Molecular Research Center, Cincinnati, OH, USA), according to the manufacturer's instructions. Reverse transcription (RT) was performed using random hexamers and ready-to-Go RT–PCR beads (Amersham Biosciences, Piscataway, NJ, USA) for nine overlapping fragments of the ATM gene, according to instructions. Long SSCP analysis was performed on the RT–PCR products, as previously described (Kukita *et al*, 1997). A suspected sample was subjected to direct sequencing using the cycling sequencing system (Applied Biosystems, Foster City, CA, USA) and run on Perkin-Elmer ABI-377 automatic sequencer.

High molecular DNA was extracted from normal and tumour tissues using standard procedures. An identified mutation was verified on the tumour DNA and then on the normal DNA (PBL) for the identification of a germ-line mutation.

Each specific missense mutation was screened in 100 matched normal ethnic group: Ashkenazim or Arabs or non-Ashkenazim, a total of 300 normal controls. The mutations were screened using SSCP analysis for each of the three exons −13, 31 and 39. Restriction analysis using *Ssp*I and *Mnl*I was used for the mutations in exons 5 and 14, respectively.

Loss of heterozygosity analysis was performed on 15 paired tumour and blood DNA samples using four microsatellite markers. Two markers were located within the 11q22–q23 locus (D11S1778, D11S1817) and two in the ATM gene (NS22 and D11S2179).

## RESULTS

Mutation analysis revealed eight alterations in seven out of 23 patients (30%) (Table 1

**Table 1:** ATM alterations in Hodgkin's lymphoma tumours and matched ethnic controls

**Exon**	**Nucleotide change**	**Amino-acid change (codon)**	**Frequency^a^ Hodgkin's lymphoma**	**Frequency controls**
5	162T → C	Y54Y	0.04	0.03
13	1784T → C	V595A	0.02	0
14,31	1810C → T/4388T → G	P604S/F1463C	0.02	0.003
39	5557G → A	D1853N	0.07	0.12

a Allelic counts.

). All of the alterations were base substitutions, and in all cases the wild-type allele was preserved.

Normal tissue was available in all seven patients and their normal DNA was screened for identified alterations. In all, the alterations were present in the germ-line. The frequency of ATM variants was analysed in samples of matched ethnic origin (Table 1). Five patients were found to have two polymorphic changes: Y54Y in exon 5 and D1853N in exon 39. Two patients of Arab origin harboured the silent change Y54Y, which was observed with similar frequency in the Hodgkin lymphoma patients and matched ethnic controls, 0.04 and 0.03, respectively. D1853N was observed in three patients of non-Ashkenazim origin, at an allele frequency of 0.07 in our Hodgkin lymphoma patients and at a frequency of 0.12 in the matched ethnic normal population.

The other alterations were P604S/F1463C in exons 14 and 31, respectively. The P604S/F1463C combined genotype was identified in one Hodgkin lymphoma of a mixed Ashkenazim/Iraqi origin and in one of the control cases. The frequency of each allele separately in the normal population was 0.015 and 0.02 for P604S and F1463C, respectively. The novel mutation V595A was observed in a Bedouin patient and in none out of 100 ethnic matched controls.

Loss of heterozygosity analysis was performed on 15 paired tumour and normal samples. In all, 12 patients were informative in at least two markers and four (33%) exhibited LOH (Table 2

**Table 2:** LOH analysis of Hodgkin's lymphoma tumors

**Patient**	**CEN**	**ATM gene**			**TEL**
**no.**	**D11S1817**	**NS22**	**D11S2179**	**Genotype**	**D11S1778**
1	+	+	+	D1853N±	+
2	–	+	+	P604S/F1463C±	+
3	+	+	+		+
4	LOH	INS	–	Y54Y±	–
7	–	−	–	D1853N±	+
8	–	+	+		+
9	+	+	–	D1853N±	–
10	+		+		–
11	–	±	+		+
12	–	+	+	Y54Y±	–
13	LOH	INS	–		+
14	+	-	–		–
15	LOH	+	+		–
17	LOH	LOH	–		LOH
18	–	–	+		–

CEN=centromeric; TEL=telomeric; −: not informative; +: informative; LOH=loss of heterozygosity; INS=microsatellite instability. ± heterozygote.

). In three patients, the region of LOH was located centromeric to the ATM gene and, in one, the LOH spanned a large region, including the markers centromeric and telomeric to the ATM gene. In addition, two patients showed genomic instability in the NS22 marker, which is located in the ATM gene.

Three out of 23 patients were studied, relapsed, and in two of them ATM missense alterations were identified. The patient harbouring the combined genotype P604S/F1463C relapsed within 12 months after diagnosis and expired 7 months later. The other patient, carrying the V595A alteration, relapsed twice, after 28 months and subsequently after additional 10 months, following autologous bone marrow transplantation.

## DISCUSSION

In this study, ATM missense variants were detected in two out of 23 (9%) childhood Hodgkin tumours. All alterations were present in the germ-line. These two patients had a more aggressive clinical course of the disease.

The P604S/F1463C genotype was reported previously in two Hodgkin's disease patients of Ashkenazi origin (Offit *et al*, 2002). It is of note that our patient was of a mixed Ashkenazi/Iraqi origin. Unfortunately, we were unable to determine the origin of the mutation, as his parents were not available for analysis. The novel mutation V595A was observed in a Bedouin patient who inherited it from his mother, and was not identified in 100 matched normal ethnic population.

In addition, we detected two polymorphic changes that have been described in previous studies. Y54Y silent change was described recently in an A-T patient who harboured two truncation mutations (Buzin *et al*, 2003). D1853N is a well-established known polymorphism that was observed at a similar frequency in cancer patients and in controls reported in other studies (Thorstenson *et al*, 2001; Offit *et al*, 2002).

Four out of 12 informative patients exhibited LOH centromeric to ATM locus, and only in one patient LOH encompassed the whole ATM region. Thus, LOH of the ATM gene is rarely involved in childhood Hodgkin disease. In molecular and cytogenetic studies of Hodgkin disease, loss of 11q was found to be a rare event (Tilly *et al*, 1991; Dohner *et al*, 1992; Ohsima *et al*, 1999). In a report utilising one marker telomeric to the ATM locus, three out of seven Hodgkin patients exhibited LOH in the this region (Hasse *et al*, 1999). All these observations may suggest that other tumour-suppressor genes that reside in 11q region may be involved in childhood Hodgkin's disease.

No protein truncation mutations were identified in our childhood Hodgkin lymphoma tumours, similar to the absence of truncation mutations reported in another study (Offit *et al*, 2002), which included both adults and children. Moreover, no truncating germ-line ATM mutations were detected in 52 survivors of Hodgkin's disease, adults and children, who developed secondary neoplasms (Nichols *et al*, 1999).

In studies of ATM gene involvement in adult sporadic lymphoid malignancies, missense and truncation mutations, both somatic and germ-line origin, were identified. In T-PLL, missense mutations clustered to the PI3-K region (Vorechovsky *et al*, 1997), while in B-CLL (Stankovic *et al*, 1999), MCL (Camacho *et al*, 2002) and DLBCL (Gronbaek *et al*, 2002) missense mutations spread across the whole gene. Missense variants identified in the present study were located outside the PI3-K region, similar to results in the study by Offit *et al* (2002). The only alteration in a known domain is the F1463C variant that resides in the FAT region. In addition, the majority of cases of adult lymphoid malignancies such as MCL and B-CLL show biallelic inactivation of the ATM gene. It is different in children with ALL, in whom single missense mutations with no LOH involvement were reported (Pause *et al*, 2003). In our study of childhood T-cell ALL, truncation and missense mutations were detected, also without LOH involvement, and we could not identify any genetic alteration in T-cell non-Hodgkin's lymphoma (in press). This could suggest a dominant-negative mechanism, as was demonstrated by Scott *et al* (2002); some missense mutations that reside outside the PI3-K region have an effect on ATM downstream targets by a dominant-negative mechanism. In a more recent study by Oguchi *et al* (2003), the dominant-negative effect of a missense mutation located in the PI3-K domain was demonstrated in a childhood acute leukaemia patient with MLL rearrangement.

Our results indicate that missense variants of the ATM gene occur in Hodgkin's disease in children (9%), but at a low frequency, and thus do not play a major role in the oncogenesis of the disease in our childhood population in Israel. On the other hand, observation of LOH outside the ATM locus may indicate the involvement of other tumour-suppressor genes in this disease. The presence of missense variants was associated with a more aggressive disease, and further investigation of these substitutions should clarify whether they have any functional effect on the oncogenesis of Hodgkin lymphoma.
